# 

**Published:** 1884-06

**Authors:** 


					﻿THIRTYXFIRST YEAR.

JOURNAL
OF

Guaranteed Circulation 200,000 Copies in 12 Months.
E. H. GIBBS, A.M., M.D., Editor,
No. 21 CLINTON PLACE EIGHTH STREET NEW YORK.
TERMS: $1 a Year.
Single numtier, 10 ctsJ
				

